# Application of Two Novel Acoustic Emission Parameters on Identifying the Instability of Granite

**DOI:** 10.3390/e24060750

**Published:** 2022-05-25

**Authors:** Zhenxing Ji, Peihua Jiang, Haiyang Yi, Zhuang Zhuo, Chunyuan Li, Zhide Wu

**Affiliations:** 1 School of Mine Safety, North China Institute of Science and Technology, Langfang 065201, China; ji99742294@gmail.com; 2 School of Architecture Engineering, North China Institute of Science and Technology, Langfang 065201, China; lichunyuan@ncist.edu.cn; 3 School of Safety Engineering, North China Institute of Science and Technology, Langfang 065201, China; zhuo@ncist.edu.cn; 4 Research Institute of Petroleum Exploration and Development, Beijing 100083, China; wuzhide69@petrochina.com.cn

**Keywords:** information entropy, correlation dimension, acoustic emission, rock instability

## Abstract

The issue of monitoring and early warning of rock instability has received increasing critical attention in the study of rock engineering. To investigate the damage evolution process of granite under triaxial compression tests, acoustic emission (AE) tests were performed simultaneously. This study firstly introduced two novel parameters, i.e., the coefficient of variation (CoV) of the information entropy and correlation dimension of the amplitude data from the AE tests, to identify the precursor of the failure of granite. Then the relationship between the changes in these parameters and the stress-time curve was compared and analyzed. The results of this study show that: (1) There is a strong correlation between the CoV of the information entropy and the failure process of granite. The granite failed when the CoV curve raised to a plateau, which could be used as an indicator of rock instability. (2) The fluctuation of the correlation dimension indicates the different stages during the loading process, i.e., the initial compaction stage, the linear elastic stage, the yield stage, and the failure stage. Each stage contains a descending and a rising process in the correlation dimension curve, and the exhibited starting point or the bottom point at the correlation dimension curve could be selected as the indicator point for the rock instability. (3) The combined analysis of the Information entropy and Correlation dimension can improve the accuracy of rock instability prediction. This study provides new insights into the prediction of rock instability, which has theoretical implications for the stability of subsurface engineering rock masses.

## 1. Introduction

The investigation of confining rock stability is an increasingly important topic in the study of safe construction in underground engineering [1,2]. On account of forecasting the early danger of engineering instability, knowledge of the damage process of confining rock during stress loading is of significant importance to the theory of failure precursor detection.

Extensive studies have been performed to investigate the damage evolution mechanism of rocks based on triaxial compression tests [3,4,5,6,7]. In general, the damage process of rocks under triaxial compression can be categorized into four stages, i.e., the initial compaction stage, the linear elastic stage, the yield stage, and final broken stage [8,9,10,11]. Correspondingly, a number of theoretical models were proposed to characterize different stages of the damage evolution process of rocks [12,13,14]. According to the study of Ahmed [15] and Gong [16], the essence of rock instability is the accumulation, transformation, and external release process of internal elastic strain energy. The study of identifying and predicting rock instability has gradually become a critical issue in the study of underground engineering stability.

As an accompanying phenomenon of rock break and failure, acoustic emission (AE) technology can be used to monitor the stress wave transmission during the energy release process. Therefore, AE data is widely used for the real-time monitoring of the crack evolution process of rocks [17,18,19,20]. Related studies have shown that the AE signal carries a considerable amount of original fracture information, making it suitable to be a useful information carrier that reflects pore changes inside the rock [21,22,23]. The occurrence of rock mass instability event is often accompanied by great changes in AE parameters such as the increase of AE counts and energy [24]. For this reason, tons of studies related to the application of AE technology in the detection of precursor information of rock failure have been performed.

As previously stated, AE is a phenomenon in which elastic waves are emitted as a result of irreversible changes in rock structure caused by numerous external and internal physical forces [25]. Therefore, the AE waveform contains the original rockburst signal characteristics such as amplitude, counts, energy, and frequency. A number of studies have explored the potential of using the AE original signal characteristics to identify the damage process of rocks. For example, Zhao et al. [26] and Moradian et al. [27] found that the peak amplitude increases and reaches the maximum value until failure during the rock deterioration process. Dong et al. [28] found that AE event rate and wave velocity can both reflect the deterioration process of rocks under triaxial compression. He et al. [29] successfully simulated the rock burst process and found that AE count and energy increases rapidly when rock burst occurs. Besides, he also reported that the spectrum characteristics of AE signals generated by tensile failure and shear failure were different. Wang et al. [30] found that AE signals would be relatively less before rock mass instability.

However, difficulties arise when an attempt is made to use the basic parameters directly without processing for monitoring and early warning of the rock instability. One major drawback of using the basic parameters is the difficulty of distinguish the parameter characteristics of rocks with different lithology and occurrence conditions. Another problem of the AE basic parameters is the numerous environmental noises in practical application [31]. Given these problems, lots of research has been done by processing the characteristic parameters of AE information before analyzing. One of the processing method is to use fractal theory to analyze the variation of AE original signal characteristics. Fractal theory can describe the chaotic behavior in nature. In 1999, Xie et al. [32] developed the notion of fractal rock mechanics by successfully combining fractal theory geometry and rock mechanics. Based on the number-radius relation of fractals, Xie and Pariseau [33] found that rock fracture has fractal characteristics, and he analyzed the mechanism of rock burst from the perspective of rock damage and fractal theory. Qiao and Cao [34] and Li et al. [35] applied fractal dimension into the characterisation of AE signals to identify crack identification and quantify crack damage. As one kinds of the fractal dimension, correlation dimension was also been used to process the AE signal characteristics. For example, Li et al. [36] and Zhang et al. [37] calculated the correlation dimension of the AE count and energy during true triaxial compression tests, pointing out that the gradual reduction of correlation dimension can be used as the precursor information of coal sample instability. Gao et al. [38] discovered that the fractal dimension of AE count was relatively small during the early loading stage and reached a maximum value after the stress peak, and then followed by a cliff drop. According to the processed AE parameter named *b* value and the AE spectrum characteristics, Chen et al. [39] divided AE peak frequency into three frequency bands and found that LH and HH signals usually came before rock mass instability, which may be used to predict rock mass instability. Although the application of fractal theory achieved success in showing the relationship between AE signals and the damage process, current research failed to locate a clear turning point that indicates the failure of rocks.

Rock instability is a nonlinear process accompanied by irregular bursts of energy, which can be characterized by chaos-related indicators such as entropy. Entropy is a physical quantity that represents the degree of chaos in the system, and its essence is the description of different energies in the phase space system. Wang et al. [40] pointed out that information entropy can be used to predict rock instability, and the entropy of dominant frequency decreases significantly after reaching the peak. Ren et al. [41] studied the critical characteristics of shale failure by using information entropy and showed that in the process of triaxial compression, the fractal dimension of the RA value of shale showed a pattern of “rising-dipping-fluctuation”. The acoustic emission information entropy also has a strong relationship with rock instability, and the situation where the system has uncertain energy can be studied from the nature of energy fluctuation. However, their research is mostly focused on the application of information entropy in AE count, frequency, and energy. As an important basic signal characteristics in AE, the application of information entropy and correlation dimension in the amplitude was not well investigated. Therefore, it is necessary to investigate the information entropy change of AE amplitude and explore the full applicability of entropy theory in rock instability.

This paper aims to applying two parameters based on AE tests to identify the precursor information of rock failure and instability. The relation between these two parameters, i.e., the coefficient of variation (CoV) of information entropy and the correlation dimension of the AE amplitude, with the rock deterioration process was investigated. In this study, a triaxial compression experiment of granite was carried out, and the acoustic emission amplitude information throughout the rock loading process was recorded in Section 2. In Section 3, the CoV of the information entropy and correlation dimension of the amplitude was calculated and analyzed to evaluate the potential of using new processed parameters for the prediction and warning of rock instability. The research conclusion is summarized in Section 4.

## 2. Materials and Methodology

### 2.1. Sample Preparation

The granite samples used in the triaxial compression test were collected at a 500 m depth underground repository in Beishan, Gansu province. The granite in this area mainly consists of plagioclase, quartz, alkaline feldspar, biotite and albite [42]. Cylinder samples with diameter of 50 mm and hight of 100 mm was prepared. The flatness error of the polished’s end sample is controlled within 0.05 mm, and the length error of the sample is less than 0.3 mm. To ensure the universality of the experimental law, four samples with different confining pressure and temperature were taken for loading tests, and the experimental data were analyzed respectively in this experiment. Specific sample parameters are shown in Table 1.

### 2.2. Experiment Apparatus and Procedure

**Figure 1:**
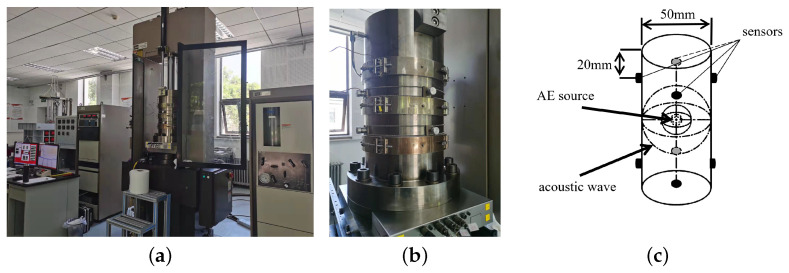
MTS815 rock mechanics test system (**a**) Overview of the test system (**b**) the triaxial cell and (**c**) layout of AE sensors.

An MTS815 hydraulic servo rockmechanics test system and a pcl-2 acoustic emission test system were used in the experiment, see Figure 1a,b. This system is capable to achieve a maximum axial bearing capacity of 4600 kN and a confining pressure of 140 MPa. In the process of applying load to the rock, the AE information measured by the AE sensor is transmitted to the host system through the preamplifier, and the storage of the AE parameters and the automatic drawing of the curve are realized.

**Figure 2:**
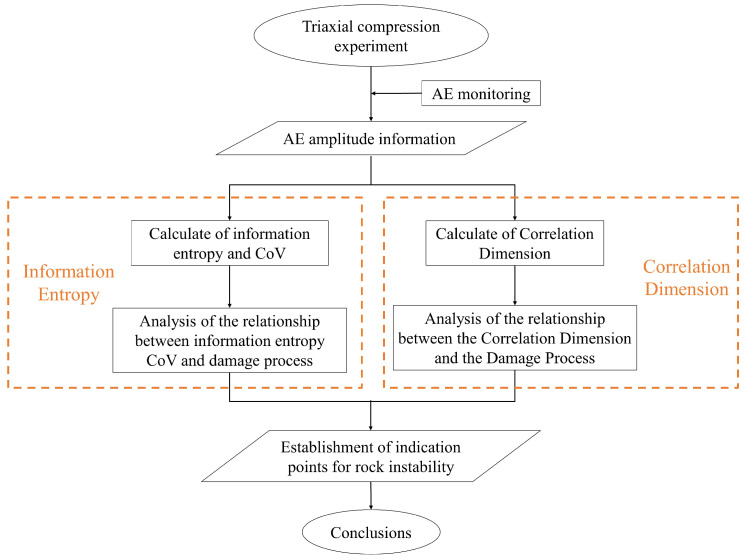
Flowchart of the techniques and steps in the procedure employed in the study.

The triaxial compression loading was controlled by axial strain and the loading rate was maintained at $2\times 10^{-6}\;\mathrm{mm}/s^{-1}$. To prevent servo interference on AE data collection, the surface of the 8 AE sensors was coated with Vaseline and attached at about 20 mm from the two ends of the coal sample, as shown in Figure 1c. The sensor response frequency is 35–1000 kHz, and the sampling frequency is set to 1 MHz. The AE signal acquisition threshold is set to 30 dB to avoid low-amplitude background noise [43]. Figure 2 shows a flowchart of the techniques and steps in the procedure employed in the study.

### 2.3. Calculation Methodology of Information Entropy and Its CoV

**Figure 3:**
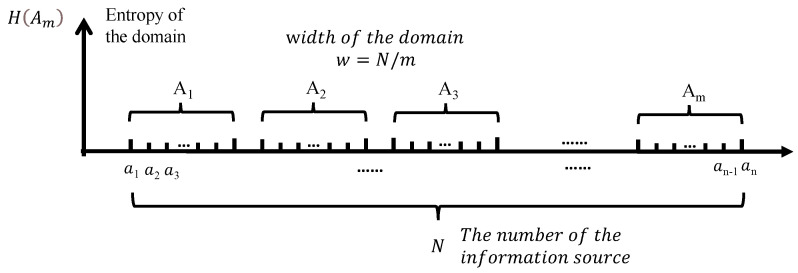
Computational theory of information entropy.

The concept of information entropy was first proposed by Shannon [44]. Information Entropy is a physical quantity that represents the disorder degree of molecular systems. It can simplify the complex information through quantitative characters. Xu [45] pointed out that the surrounding rock of underground engineering has a significant degree of nonlinearity, which is specifically reflected in the transformation of rock mass deformation and failure process from disorder to order. The magnitude of entropy means the degree of system chaos. The decrease of entropy indicates the transformation of the system from disorder to order and from irregular to regular. Since underground rock mass engineering is also a transformation from disorder to order, it is bound to produce a certain entropy change. As displayed in Figure 3, suppose there have A information sources, $A=\left\{a_{1},a_{2},a_{3}\cdots \cdots \;\cdot \;a_{N}\right\},\left(n\in N\right)$. The probability P(i) of each information source satisfies Equation (1):

(1) $$\sum_{i=1}^{n}P\left(i\right)=1$$

where 0<P(i)<1,i={1,2,3,…n}.

In the calculation process, the information sources obtained in the whole load test process were divided into several domains with the same width, the number of information sources in different domains is counted, and the occurrence probability of each information source in the domain is calculated. Then the information entropy of different domains can be expressed as:

(2) $$H\left(A_{m}\right)=-\sum_{i=1}^{n}P\left(i\right)\ln P\left(i\right)$$

where *m* is the number of the domains. $m=\left\{1,2,3,\ldots \ldots n\right\},m=\frac{n}{w}$, and the *w* is the width of the domains.

The coefficient of variation was calculated based on:

(3) C=σ/μ

where, σ represents the standard deviation and μ represents the mean value.

### 2.4. Correlation Dimension Calculation Methodology

**Figure 4:**
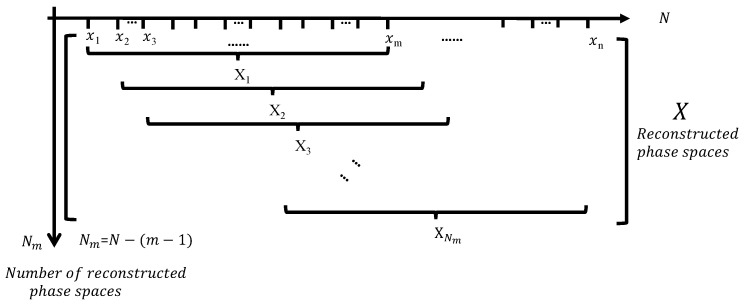
The method of phase space reconstruction of correlation dimension.

In the 1977s, Mandelbrot [46] founded the fractal theory, which established a theoretical foundation for quantitatively defining the geometric characteristics of rock structure. Xie [47] successfully combined damage mechanics with fractal geometry, established a new discipline of rock fractal theory. Among the fractal dimensions, the correlation dimension is widely used because it can better reflect the dynamic characteristics of the system [48,49]. Based on the reconstruction space theory, Grassberger and Procaccia proposed the G-P algorithm to calculate the correlation dimension D in time series [50]. Similarly, as plotted in Figure 4, the information sources generated by the AE process can form a sequence set with a capacity of *n*.

(4) $$X=\left\{x_{1},x_{2},x_{3},\ldots x_{n},\right\}$$

Take m(m<n) numbers of them as a vector set, move backward and then take *m* numbers to form another vector set. By analogy, the sequence set can form *N* vectors N=n−m+1. The calculation formula of correlation dimension can be expressed as:

(5) $$\left.\begin{array}{c} D=\lim_{r\to 0}\left(\ln C\left(r\right)/\ln r\right)\\ C\left(r\right)=\frac{1}{N^{2}}\sum_{i\neq j}^{N}H\left(r-\left|x_{i}-x_{j}\right|\right) \end{array}\right\}$$

where C(r) is Correlation functions; H(r) is Heavjiside functions, $H\left(x\right)=\left\{\begin{array}{cc} 1 & \left(x>0\right)\\ 0 & \left(x⩽0\right) \end{array};\right.$ *r* is the distance between two points in the fractal dimension, $r\left(k\right)=k\frac{1}{N^{2}}\sum_{i=1}^{N}\sum_{j=1}^{N}\left|X_{i}-X_{j}\right|$.

## 3. Results and Discussion

### 3.1. AE Amplitude Changes during the Loading Process

**Figure 5:**
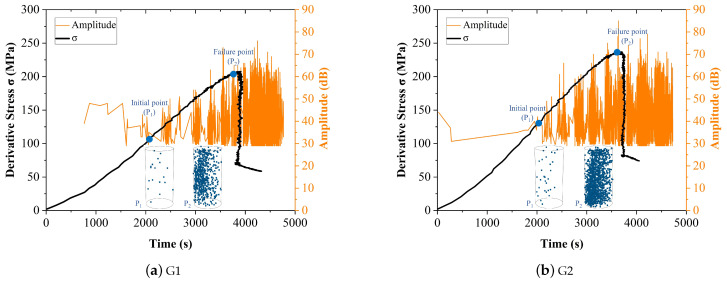

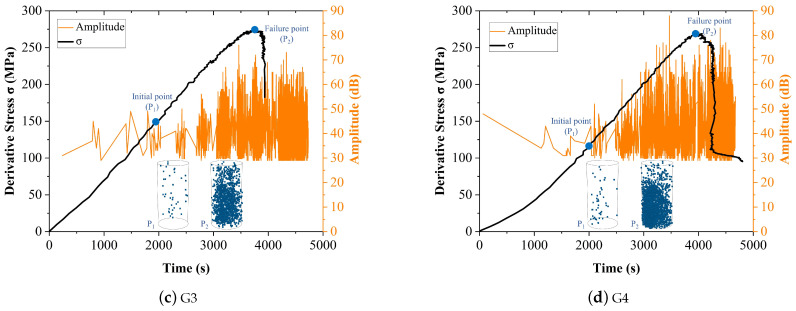
The Stress curve and AE amplitude of the samples (**a**–**d**) G1 to G4, respectively.

The changes in AE amplitude and derivative stress of the four tested samples were shown in Figure 5, the initial and failure point were marked and the AE location of these two points were drawn. It can be seen that the number of amplitude data was very small at the beginning of the initial compaction stage (approximately the first 2000 s). When the samples turned into the linear elastic stage (2000 to 3000 s), the number of amplitude data increased and the value of amplitude increased as well. The value of AE amplitude reached a maximum at the moment of rock failure (about 3700 s), and more amplitude data were generated after the failure of the samples.

Based on Figure 5, even though there is a correlation between the AE amplitude and the damage evolution process, the variation of AE amplitude is discrete and it’s difficult to locate a clear turning point that indicates the moment of failure. Therefore, it is necessary to process these data before using it as an indicator of rock instability.

### 3.2. Relationship between the Information Entropy CoV and the Damage Process

**Figure 6:**
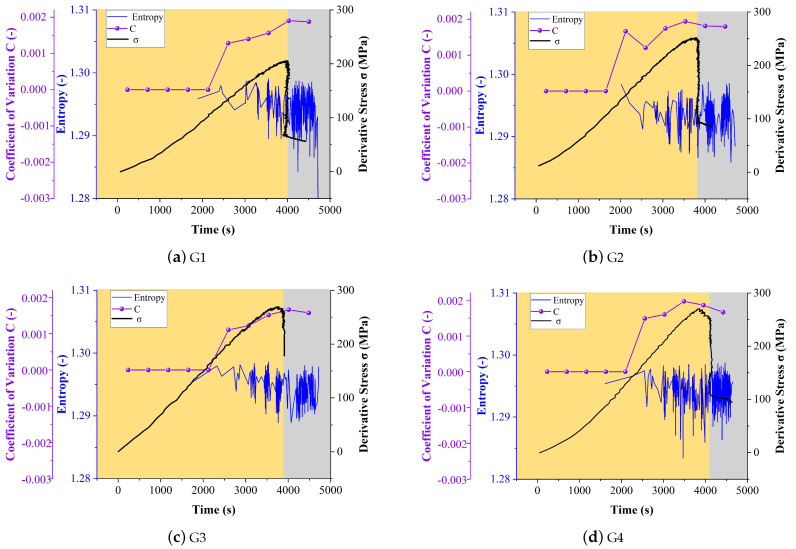
AE amplitude information entropy and CoV curves of rock samples (**a**–**d**) G1 to G4, respectively.

Figure 6 shows the calculated information entropy, the CoV of the information entropy, and the stress-time curve during the loading process. The domain width was selected as w = 20 when calculated the information entropy. Besides, the loading period was shaded by yellow and gray to represent the loading process before and after the rock failure.

It can be seen from the Figure 6 that after the rock enters the linear elastic stage, the sample is in a state of initiation and expansion of internal micro-cracks, the AE activity increases rapidly, and the entropy information begins to appear. With the propagation of the internal fracture, the rock reaches the stage of crack interaction, a large number of cracks begin to consolidate and merge, and AE activity becomes strong. The entropy decreased throughout the first two phases and reached its minimum value near the end of the stable stage, this is because AE sources go through a process of random excitation to gather around cracks in the process of rock fracture aggregation. The penetration of the cracks causes the AE source to be ordered and the information entropy to decrease. The aggregation of cracks leads to the order of the acoustic emission source, and the information entropy decreases accordingly, which is also the beginning of rock instability and failure. The fractures connected and further propagated during the stage of rock instability and failure. Because of the rock’s compression failure, a significant number of AE activities are formed, and the AE activities became irregular again. The information entropy tends to rise and fluctuate in a smaller range.

CoV is a statistic that characterizes the degree of data dispersion. According to the CoV changes of the information changes, the variation frequency of the information entropy increased during the loading process (shaded with the yellow color) and reached a plateau or slightly decreased before the failure of the rock (shaded with the gray color). This means during the first three stages, the measures of dispersion of the information entropy are becoming bigger. When the CoV hits its maximum, it indicates that the rock is beginning to fracture and begin to descend in the final broken stage. These results indicated that this parameter could be used as an indicator of predicting the fail of rock.

In summary, in the process of rock compression failure, the cracks of rock have experienced the process of initial compaction—stable development of cracks in elastic stage —aggregation and penetration of cracks in final broken stage, which is specifically manifested in the transformation from disordered to orderly and irregular to regular.The information entropy curve will fluctuate greatly during the loading process, but the overall trend decreases before the rock failure, and reaches the minimum value when the rock fails. In the post-peak stage, the AE activity of the rock is frequent and irregular, and the change of entropy shows characteristics of irregular, frequent and small fluctuations, but the overall trend is higher than that before failure.

### 3.3. Relationship between the Correlation Dimension and the Damage Process

Similar to the width of the information entropy, the selection of the dimension *m* and the length of the sample size *n* have a great influence on the calculation accuracy. A small value of *m* will lead to the incomplete expansion of the phase space, while a large value of *m* will cause the adjacent two-phase points to be too separated to calculate the fractal dimension. In this paper, the geometric invariant method is used to calculate *m*, and when *m* grows to a certain value, *D* begins to converge, indicating that the dimension *m* is the optimum dimension.

**Figure 7:**
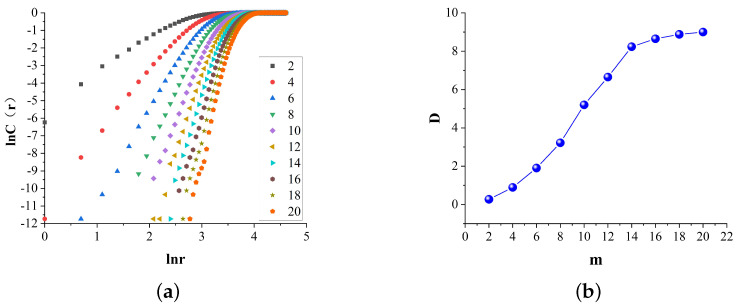
Evolution in (**a**) the r−Cr Curve with different dimensions m and (**b**) the change curve of D in different dimensions m.

Matlab compiler was used to calculate the changes of observation scale *r* and correlation function C(r). Figure 7a shows the r−C(r) curve under different dimensions *m*, and the slope of r−C(r) curve is the correlation dimension *D*. As shown in Figure 7b, *D* starts to converge when m=14, and m=14 is final taken in this paper.

**Figure 8:**
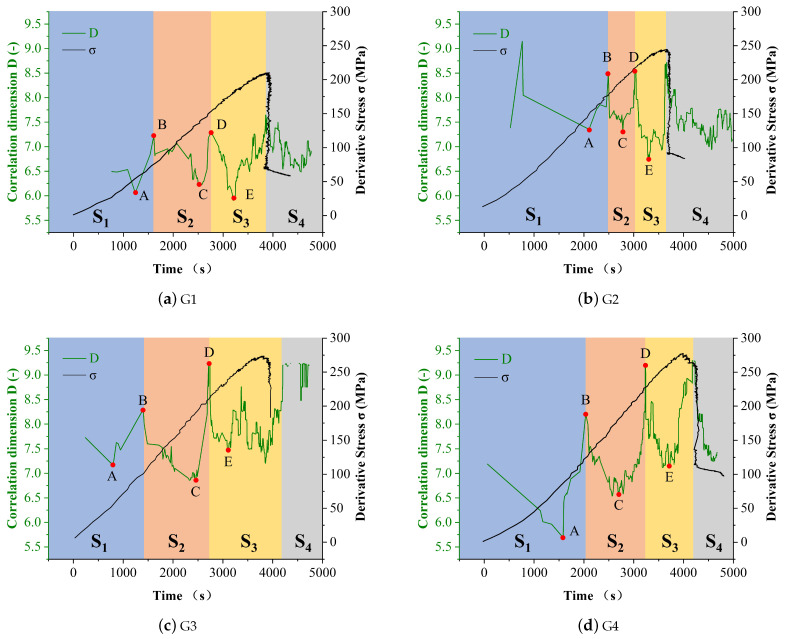
Correlation dimension curve of AE amplitude of rock samples (**a**–**d**) G1 to G4, respectively.

Figure 8 shows the correlation dimension variation curve of acoustic emission amplitude of rock in the loading process. The correlation dimension of the amplitude represents the degree of separation in location during the loading process. For example, a high correlation dimension value indicates that the newly generated crack is distributed in a large domain. In opposite, a small correlation dimension means the newly generated cracks are concentrated in a small area. According to Figure 8, correlation dimension changes during the loading process could be generally divided into four stages: the initial compaction stage, the linear elastic stage, the yield stage, and the failure stage. Each stage contains a descending process and a rising process.

For the initial compaction stage, the correlation dimension decreased at the beginning due to the closure of the internal cracks and the decrease of the voids ratio. When the rock was initially compacted, the deviation stress reached the critical crack initiation stress (point A) and micro-cracks started to generate at the weakness area. For this reason, the correlation dimension started to increase from point A.

Then the rock behaved as linear elastic property at point B. In this stage, there are two opposite effects that influence the changes in the correlation dimension. Point B is the critical crack damage point, which represents micro-cracks start to propagate on the crack tip. For this reason, the correlation dimension decreased starting from point B. In the meantime, as the internal stress increases, more micro-cracks were generated which has the effect of increasing the correlation dimension. When the effect of increasing this value is larger than decreasing it (point C), the correlation dimension starts to increase until point D.

Point D represents the critical crack interaction point, which means the propagated cracks start to connect and form transfixion cracks. For this reason, new cracks were mostly generated near the old cracks and the correlation dimension decreased from this point. When the transfixion cracks were generated at point E, more secondary cracks were generated and the rock was about to fail.

To sum up, the correlation dimension of the amplitude in the AE tests could accurately indicates the different stages of the loading process. Combined with the implication of the information entropy, the accuracy of determining the failure of rock could be guaranteed.

## 4. Conclusions

To reveal the relationship between the processed AE parameters, i.e., the information entropy and correlation dimension of the amplitude, triaxial compression experiments were carried out, and acoustic emission was monitored during the loading process. The information entropy and correlation dimension of AE amplitude about time series were calculated and compared, and the potential of using the information of these two parameters to determine the rock failure was investigated. The specific conclusions are as follows:

(1) Both the AE amplitude information entropy CoV and the correlation dimension curve under the triaxial loading state of the rock could accurately characterize the rock instability process.
(2) According to the relation between the CoV of information entropy with the different stages of the loading process, the variation frequency of the information entropy increased during the loading process and then reached a plateau or slightly decreased before the rock failure. This phenomenon could be used as an indicator of rock instability.
(3) Based on the changes in correlation dimension with the different stages of the loading process, it can be seen that each stage (the initial compaction stage, the linear elastic stage, the yield stage, and the failure stage) was well characterized by the correlation dimension. The starting point (D) or the bottom point (E) could be selected as the indicator point for the rock instability.
(4) The method of combining the signals of information entropy CoV and correlation dimension could achieve a good results in identifying the precursor information of rock failure and instability.

## Figures and Tables

**Table 1:** The confining pressure and temperature of the sample.

Sample ID	Confining Pressure/MPa	Temperature/°C
G1	15	50
G2	20	50
G3	20	25
G4	25	70

## Data Availability

The data supporting this study are included in the paper.
